# Traditional Chinese medicine (*Xiaoai Jiedu Decoction*) as an adjuvant treatment for prevention new colorectal adenomatous polyp occurrence in post-polypectomy: Study protocol for a randomized controlled trial

**DOI:** 10.1097/MD.0000000000016680

**Published:** 2019-08-02

**Authors:** Qing Zhou, Yu-Gen Chen, Jun Xiao, Ting-ting Chen, Jian-rong Liu, Wan Zhou, Wei-zhong Li, Yan Wang, Bei-ping Zhang, Jiang lin, Fan-dong Meng, Biao Gong, Guo-ying Zheng, Shu-tang Han, Hai-bo Cheng

**Affiliations:** a Affiliated Hospital of Nanjing University of Chinese Medicine; b Nanjing University of Chinese Medicine; c The First Affiliated Hospital With Nanjing Medical University, Nanjing, Jiangsu; d Guangdong Province Hospital of Traditional Chinese Medicine, Guangzhou, Guangdong; e Longhua Hospital Shanghai University of Traditional Chinese Medicine, Shanghai; f Beijing Friendship Hospital, Beijing; g Shuguang Hospital Shanghai University of Traditional Chinese Medicine; h Changhai Hospital of Shanghai, Shanghai, China.

**Keywords:** Chinese herbal medicine, colorectal adenomatous polyp, randomized controlled trial, *Xiaoai Jiedu decoction*

## Abstract

**Background:**

Colorectal Adenomatous Polyp (CAP) was one precursor of colorectal cancer (CRC) and having a high chance of developing into CRC. There was a lack of conclusive chemoprevention evidences to prevention new CAP occurrence in post-polypectomy. *Xiaoai Jiedu Decoction*, Chinese National Medical Professor (Zhou Zhongying)'s experience formula, has been used to treat new CAP occurrence in post-polypectomy from the 20th century in China. However, clinical research of *Xiaoai Jiedu Decoction* in the treatment of CAP recurrence was lack. We design this study to evaluate the efficacy and safety of *Xiaoai Jiedu Decoction* in the treatment of new CAP occurrence in post-polypectomy on colonoscopy.

**Methods/Design:**

A randomized, controlled, blind and multicenter trial to evaluate the efficacy and safety of *Xiaoai Jiedu Decoction* is proposed. CAP patients (after complete polypectomy under colonoscopy) will be randomly assigned into *Xiaoai Jiedu Decoction* group and *Xiaoai Jiedu Decoction* mimetic agent group. Patients will receive 6-course treatments and a 2-year follow-up. Follow-up colonoscopy will be anticipated to perform in 1 and 2 years after the baseline examinations. The primary outcome measure is the new CAP occurrence in 1 and 2 years. The secondary outcome measure is the occurrence of advanced adenoma in 1 and 2 years.

**Discussion:**

This study will provide objective evidences to evaluate the efficacy and safety of *Xiaoai Jiedu Decoction* as an adjuvant treatment for new CAP occurrence in post-polypectomy.

**Trial registration:**

NCT03616444.

## 1 Introduction

It was widely accepted that colorectal adenomatous polyp (CAP) was well-known precursor of colorectal cancer (CRC).^[1]^ CAP constituted over 95% of CRC.^[2]^ In a recent study,^[3,4]^ Up to 30% of average risk asymptomatic individuals 50 years or older had at least one colorectal adenoma and one-third of young individuals, including in their 30 seconds and 40 seconds had colorectal polyps. Unlike with more inaccessible cancers, early detection and prevention of CAP can be achieved by screening with colonoscopy.^[5]^ Colonoscopy was the gold standard for CAP detection.^[6]^ Colonoscopy and early treatment of CAP was thought to be associated with about 80% reduction in CRC incidence.^[1]^ Based on the American Cancer Society guidelines,^[7]^ nearly half the population will be counseled to undergo a colonoscopy from 40 years old based on a positive family history of adenoma.

Unfortunately, surveillance colonoscopic screening was underutilized.^[8]^ Following their detection and removal, the recurrence CAP and CRC rate was still relatively high.^[9]^ Waye et al^[10]^ showed 56% of patients following adenoma removal on colonoscopy had further adenomas on repeat examination at 1 year. Pabby et al^[11]^ demonstrated that 53.8% of interval cancers were either secondary to incompletely resected adenomas or to missed cancer on prior colonoscopy. Therefore, the use of adjuvant chemoprevention strategies to complement surveillance screening may have a potential to further reduce CRC morbidity and mortality among those CAP.^[12]^

*Xiaoai Jiedu Decoction*, Chinese National Medical Professor (Zhou Zhongying)'s experience formula, a kind of Chinese herbal medicine, listed in Table 1, has been used to treat CAP recurrence in post-polypectomy from the 20th century in China. However, it was still necessary to prove the efficacy and safety of this Chinese medicine. The aim of the present study is to evaluate *Xiaoai Jiedu Decoction* in the treatment for prevention new CAP occurrence in post-polypectomy using a randomized, controlled, blind and multicenter trial among officially registered Chinese colorectal consultants, fellows and residents in China.

**Table 1:**
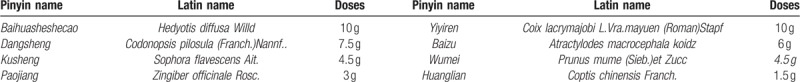
Standard formulation of *Xiaoai Jiedu Decoction*.

## 2 Methods/design

### 2.1 Design

**Figure 1:**
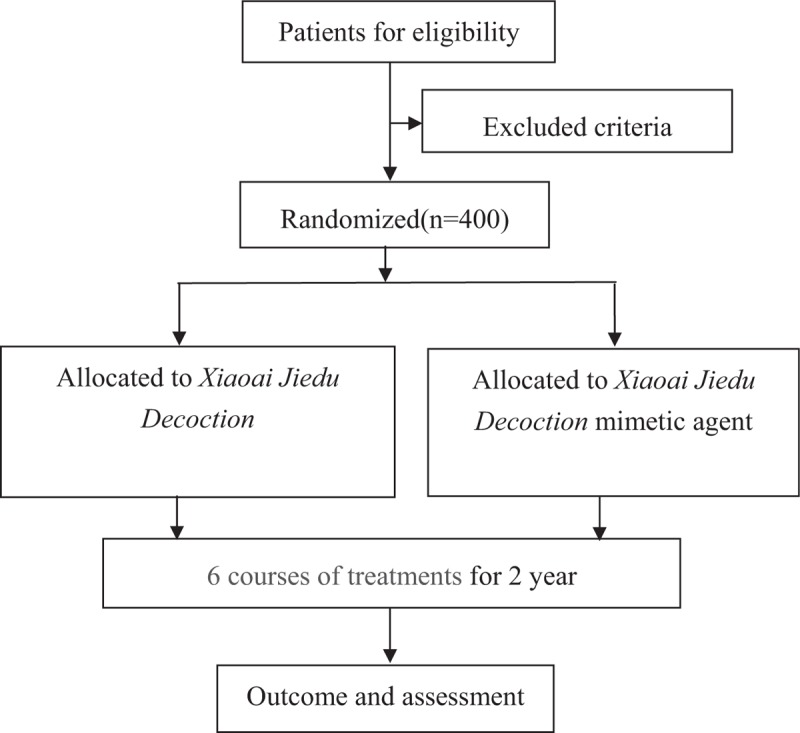
Study flow chart. The flow chart of enrolment, allocation, intervention, and assessment.

This study is designed as a randomized, controlled, blind, and multicenter trial. This study is registered with ClinicalTrials.gov (NCT03616444). Trained researchers introduce the trial to patients, give them information sheets and consent forms. All patients have to give their written informed consents prior to enrolment. The study's flow chart is shown in Figure 1.

### 2.2 Ethics

The trial protocol is conducted in accordance with the Good Clinical Practice Guidelines and the *Declaration of Helsinki* (2008).^[13]^ Ethics approval has been obtained from the ethic committee of Affiliated Hospital of Nanjing University of Chinese Medicine (Number 2018NL-067–03 ). Written informed consent will be obtained from each patient.

### 2.3 Recruitment

A total of 400 Chinese patients who fulfill the screening criteria will be recruited at seven hospitals in China:

1. Affiliated Hospital of Nanjing University of Chinese Medicine, will recruit 100 patients through posters,
2. Guangdong Province Hospital of Traditional Chinese Medicine, will recruit 60 patients through posters,
3. Longhua Hospital Shanghai University of Traditional Chinese Medicine, will recruit 48 patients through posters,
4. The First Affiliated Hospital With Nanjing Medical University, will recruit 48 patients through posters,
5. Beijing Friendship Hospital, will recruit 48 patients through posters,
6. Shuguang Hospital Shanghai University of Traditional Chinese Medicine, will recruit 48 patients through posters,
7. Changhai Hospital of Shanghai, will recruit 48 patients through posters.

### 2.4 Sample size

According to the literature search, new CAP occurrence in post-polypectomy was used as the scoring criteria for the treatment of polypectomy under colonoscopy. The new CAP occurrence rate under colonoscopy treatment was about 30% (P_0_), and the new CAP occurrence after the intervention with *Xiaoai Jiedu Decoction* was about 16.4% (P_1_). According to the 1:1 parallel control principle, for a 2-sided significance level of .05 and power of 90% (α = 0.05, β = 0.1), the sample size is calculated using the formula: 
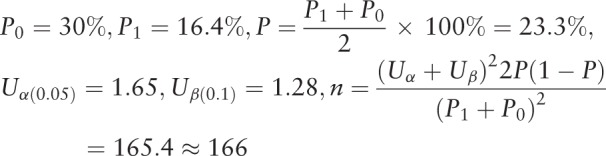


Considering a 20% loss to follow-up, the sample size is 400 cases (n = 200 in each group).

### 2.5 Randomization

Block randomization was used. Stratification was carried out according to the center. With the help of SAS 9.4 statistical software, the random number table of central coding was generated for a given number of seeds. The subjects were randomly divided into *Xiaoai Jiedu Decoction* group and *Xiaoai Jiedu Decoction* mimetic agent group. An independent person (Wan Zhou), who is not involved in observation or assessment of the patients possess the computer-generated randomization sequence. The randomization procedure will be conducted by research assistants using an online computerized randomization system (https://sci.medroad.cn/).

### 2.6 Blinding

This trial is a blind trial, divided into *Xiaoai Jiedu Decoction* group and *Xiaoai Jiedu Decoction* mimetic agent group. The double levels of blinding are sealed separately, and given to the leader and the sponsor of the clinical research. Each hospital receives an emergency letter, along with the test drug, properly preserved until the end of the trial. Treatments are blinded to the patients and investigators (including statisticians) until the entire study is completed.

Code-breaking should occur only in the case of serious adverse events happen or further intervention of the patient needs to know the actual medication situation, with the permission of the person in charge of the research center, and a report should be submitted to the leader of the trial within 24 hours.

### 2.7 Eligibility criteria

Inclusion criteria:

(1) Subject is between 18 and 70 years of age;
(2) Subject is diagnosed by pathologically proven colorectal adenomatous polyp (that is tubular adenoma, tubulovillous adenoma and villous adenoma);
(3) Subject had completeness colorectal adenoma removed within 30 days before enrollment, had no remaining polyps after a complete colonoscopy;
(4) Subject were anticipated to undergo a 2-year colonoscopy follow up examination;
(5) Consent has been given;
(6) Subject agreed to avoid taking study agents outside the trial.

Exclusion criteria:

(1) Subject is younger than 18 or older than 70 years old at the time;
(2) Subject had a history of inflammatory bowel disease, or a history of colorectal cancer;
(3) Subject had been diagnosed or had a history of any cancer;
(4) Subject had familial adenomatous polyposis, or Hereditary non-polyposis colorectal cancer syndrome;
(5) Subject has other hereditary cancer syndromes including but were not limited to Peutz-Jeghers Syndrome, MYH-Associated Polyposis, Gardner's Syndrome, Turcot's Syndrome, Cowden's Syndrome, Juvenile Polyposis, Cronkhite-Canada Syndrome, Neurofibromatosis and Familial Hyperplastic Polyposis;
(6) Subject was contraindication to biopsy;
(7) Subject's bowel preparation quality was graded as poor^[14]^ (presence of semisolid stool that could not be washed away, and <90% of surface seen);
(8) Subject had incomplete colonoscopy procedure;
(9) Subject's histopathology showed the polyp were hyperplastic polyp, or serrated adenoma;
(10) Subject's polyp had infiltrated into muscularis propria, or suspected deep submucosal infiltration;
(11) Subject had accompanied by severe liver, kidney, heart, brain, or lung dysfunction;
(12) Subject is taking calcium, non-steroidal anti-inflammatory drugs, or vitamin D.
(13) Subject is pregnant and lactating women at the time;
(14) Subject will plan pregnancy during this study;
(13) Subject was allergic to test drugs and their ingredients;
(14) Subject had inability to understand the nature of the study and follow the doctor's recommendations.

### 2.8 Test drugs

Test drugs are *Xiaoai Jiedu Decoction* and *Xiaoai Jiedu Decoction* mimetic agent, provided by Tianjiang Pharmaceutical Group Co. Ltd, Wuxi, China. The mimetic agent has the same shape, size, taste, color, package, and Lot number.

## 3 Interventions

### 3.1 Liang-Xue-Di-Huang granule group

Subjects in the *Xiaoai Jiedu Decoction* granule group will be treated with *Xiaoai Jiedu Decoction*.

**Figure 2:**
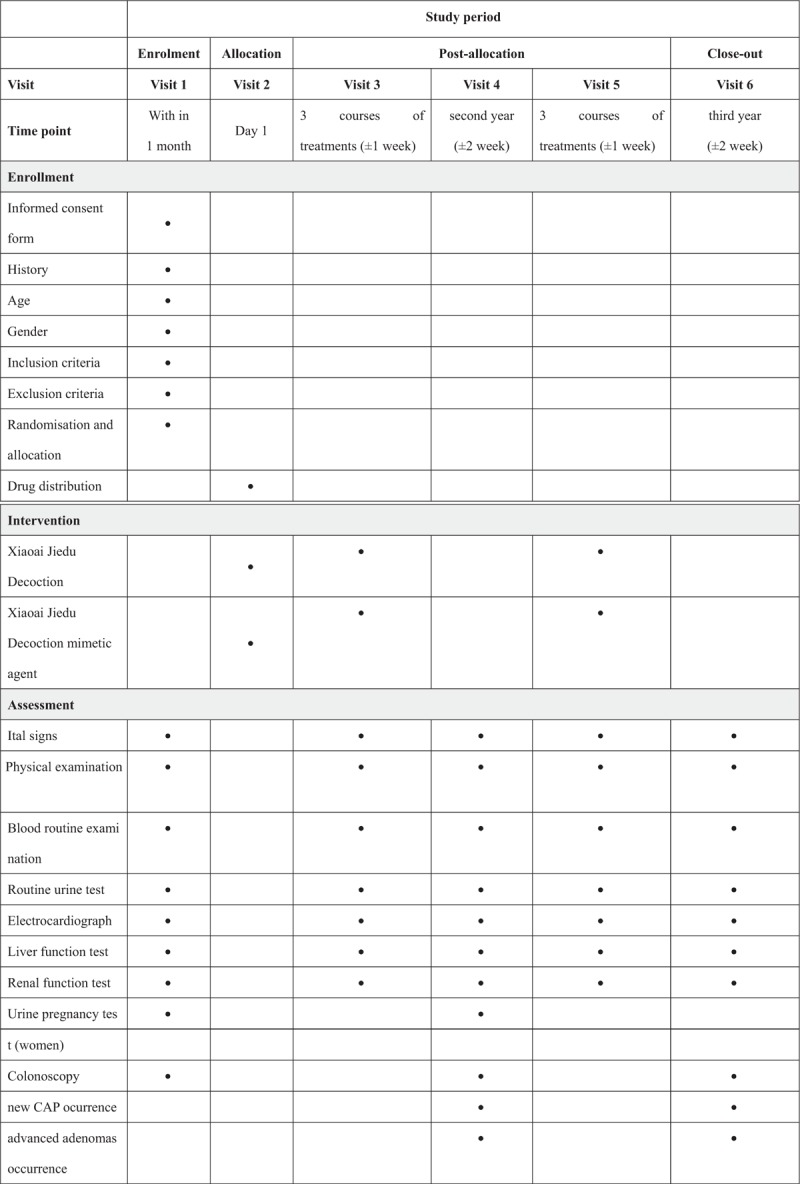
Study schedule for patients. After the enrolment and allocation, participants will take 6 courses of treatments in 2 years. The time-points of assessment are shown in the schedule.

Subjects will take one *Xiaoai Jiedu Decoction* per time, 2 times a day, be took 1 hour after lunch and dinner meals. One course of treatment will take 28 days in 1 month, and 2- to 3-day rest. In the first year, subjects will take 3 courses of treatments after signed the informed consents. In the second year, subjects will take the other 3 courses of treatments after the repeat colonoscopy examination. The course of treatment will last 6 courses of treatments for 2 years, unless there is a loss of follow-up. Subjects will be contacted by telephone every one month and queried regarding adherence to study agents, illnesses, medication and supplement use. The assessment that needs to be performed at visit is listed in Figure 2.

### 3.2 *Xiaoai Jiedu* Decoction mimetic agent group

*Xiaoai Jiedu Decoction* mimetic agent group's treatments and measurements will be in accordance with the *Xiaoai Jiedu Decoction* group.

### 3.3 Colonoscopy procedures

(1) All colonoscopies will be performed by experienced endoscopists, who have passed the unified standardized training and authorization using an EVIS LUCERA CV-260 colonoscope (Olympus Medical Systems, Tokyo, Japan);
(2) Colonoscopy should be performed under conscious sedation with intravenous midazolam and pethidine;
(3) Quality of bowel preparation will be graded as good^[14]^ (no or small volume of clear liquid, and >95% of surface seen);
(4) All subjects will received 4L/min oxygen via a nasal cannula throughout the procedure;
(5) Blood pressure will be measured every 5 minutes;
(6) Pulse oximetry and electrocardiography will be used;
(7) In order to describe the exact location of the polyps, the colon was divided into 6 sections: cecum, ascending colon, transverse colon, descending colon, sigmoid colon, and rectum. When a polyp was detected, the nurse helped perform the biopsy for histological examination, while the staff assistant recorded the location, size, and morphological features based on the Paris classification of polyps;^[15]^
(8) All polyps will be obtained and sent to the Pathology Department for histopathological examination;
(9) Withdrawal time should be greater than 6 minutes;
(10) All pathologists had board certified;
(11) Endoscopists and pathologist were blinded to history of study subjects;
(12) After colonoscopy, the patients will be disconnected from electronic monitoring and transferred to the recovery ward provided they had gained an adequate level of consciousness.

### 3.4 Outcome measures

The primary outcome measure of this study is the new CAP occurrence in 1 and 2 years. The Secondary outcome measure is the occurrence of advanced adenomas^[1]^ (defined as those with cancer, high-grade dysplasia, more than 25% villous features, or an estimated diameter of at least 1 cm as assessed by the endoscopist), and high-risk findings (any advanced adenoma and/or >2 adenomas) in 1 and 2 years.

### 3.5 Safety evaluation

A blood routine examination, routine urine test, liver function test, renal function test, electrocardiograph, and urine pregnancy test (women only) will be administered for safety outcomes, which will be monitored before clinical intervention, after 3 courses of treatments in the first year, before the second year of clinical intervention, and after 3 courses of treatments in the second year.

### 3.6 Data management

Information from the clinical examination, as well as evaluation of treatment efficacy, will be recorded in each patient's case report form (CRF). The study record is the source document of clinical study subjects and should be kept in the hospital. Each center will design designated personnel to be the electronic CRF input staff. Upon completion of each subject observation, the investigator will promptly submit the study record to the CRF inputter. The electronic CRF encoder must review whether the project record of the study notes is complete and report on time.

### 3.7 Adverse events

All adverse events, including toxicity and side effects, such as gastrointestinal reaction, liver damage, and renal failure will be recorded and graded in detail throughout the study. When a severe adverse event occurs, researchers will provide every necessary treatment, and report the adverse event to the ethic committee of Affiliated Hospital of Nanjing University of Chinese Medicine.

### 3.8 Statistical analysis

Frequency, median, and mean ± standard deviation of new CAP occurrence in post-polypectomy, and the occurrence of advanced adenomas will be used for descriptive statistics. The statistical analysis will be performed using SAS 9.4. *P* < .05 is considered statistically significant.

### 3.9 Discuss

CAP was precursor to CRC, and the goal of primary prevention is to remove them before they progress to invasive cancer. Although colnoscopy has been considered one of the most effective screening modalities, colonoscopy has some limitations. CAP can still occur before the expected surveillance interval after colonoscopy.^[16]^ In Hong Kong, the overall CAP-3 year recurrence rate was 7.9%.^[17]^ Therefore, it is not surprising that researchers have focused on efforts to develop chemopreventive strategies as an adjunct therapy to CAP recurrence. However, despite compelling data showing that a large number of chemopreventive agents show promise in CAP models, no current chemopreventive treatments can completely prevent CAP recurrence. Clinical studies have yielded conflicting results. In previously-reported randomized trial, there was no overall efficacy of calcium and or vitamin D3 against CAP recurrence.^[18]^ Protective effect of non-aspirin nonsteroidal anti-inflammatory drugs on CAP have been documented in previous systematic reviews.^[19–21]^ However, cardiovascular safety and risk of serious bleeding events hampered the acceptance of these strategies for secondary prevention of CRC.^[22]^ Therefore, the development of new chemopreventive drugs is particularly urgent for patients with CAP.

Traditional Chinese medicine (TCM) is an important component of complementary and alternative medicine, and has been developed in Asian countries, especially in China. *Xiaoai Jiedu Decoction*, Chinese National Medical Professor (Zhou Zhongying)'s experience formula, was used as an adjuvant treatment of CAP recurrence after polypectomy from the 20th century in China. Over this period, accumulating experimental data supports *Xiaoai Jiedu Decoction* to intervene in complicated molecular pathways underlying the pathogenesis of CAP and CRC. From Professor (Zhou Zhongying)'s theory, the carcinogenesis of CAP was related to the Pi-Xu (spleen-Qi deficiency), Shi-Re (damp-heat) and Ai-Du (toxicity accumulation). In his prescription, *Coix lacrymajobi L.Vra.mayuen (Roman) Stapf* (Yiyiren) and *Hedyotis diffusa Willd (Baihuasheshecao)* were the *Jun* (emperor) components in *Xiaoai Jiedu Decoction*. *Coix lacrymajobi L.Vra.mayuen (Roman) Stapf* (Yiyiren),which was first recorded in Sheng Nong's herbal classic, have been used for digestive system diseases from the 2nd century BC in China with good effects (invigorating spleen, replenishing qi, clearing damp) and few adverse events. *Hedyotis diffusa Willd (Baihuasheshecao)* which was recorded in Chinese Pharmacopoeia, had the effects of clearing heat and promoting diuresis. In vivo and in vitro studies^[23]^ have shown that *Hedyotis diffusa Willd (Baihuasheshecao)* improved the digestive tumor remission. *Codonopsis pilosula (Franch.)Nannf.* (*Dangsheng*), and *Atractylodes macrocephala koidz* (*Baizu*) were the *Chen* (minister) components, synergized with Jun to strengthen its therapeutic effects. Radix Codonopsis (*Dangsheng*) recorded in Sheng Nong's herbal classic, was often included into the formulas to treat Pi-Xu (spleen-Qi deficiency) and tumors patients, and has been approved by Chinese State Food and Drug Administration. *Atractylodes macrocephala koidz* (*Baizu*) improved the functions of gastrointestine and suppressed tumor progression. The molecular mechanism of suppress tumor progression partially via resolution of inflammatory environment.^[24]^ The *Zuo* (assistant) components, *Sophora flavescens Ait.* (*Kusheng*), *Prunus mume (Sieb.)et Zucc* (*Wumei*), and *Zingiber officinale Rosc* (*Paojiang*), cleared heat, strengthen the body to remove Ai-Du, eliminated possible adverse effects of the *Jun* and/or *Chen* components. The pharmacological activities of *Zingiber officinale Rosc* (*Paojiang*) were mainly attributed to its active phytocompounds 6-gingerol, 6-shogaol, zingerone beside other phenolics and flavonoids, which were known to have anti-oxidant and anti-inflammatory properties.^[25]^ The *Shi* (courier) component was *Coptis chinensis* (*Huanglian*). Berberine was a main component of *Coptis chinensis* (*Huanglian*). Modern medicine has confirmed^[26]^ that berberine suppressed the development of tumor cells through the inhibition of tumor cell growth and the induction of apoptosis and cell cycle arrest.

Although TCM has been clinically practiced for thousands of years, and has variety of herbal medicine, most Chinese herbal medicine products do not possess up-to-date data regarding their safety and modern scientific evidence for their claimed clinical uses. TCM safety research is becoming more standardized and is gradually aligning with international standards. Therefore, a randomized, controlled, blind and multicenter trial will be helpful for further prove to *Xiaoai Jiedu Decoction* as an adjuvant treatment of new CAP occurrence in post-polypectomy.

### 3.10 Trial status

The protocol version number is No. 5 and the date is May 09, 2019. At the time of manuscript submission, patient recruitment for the trial is on-going. The clinical study was begin from Dec, 2018 and end in Dec, 2021. A total of 400 patients will be recruited in this clinical study.

## Author contributions

**Conceptualization:** Wan Zhou.

**Formal analysis:** Wan Zhou.

**Investigation:** Ting Ting Chen, Jian-Rong Liu, Wei-Zhong Li, Yan Wang, Bei-Ping Zhang, Fan-dong Meng, Biao Gong, Guo-Ying Zheng, Jiang Lin.

**Project administration:** Hai-bo Cheng.

**Supervision:** Jun Xiao, Shu-Tang Han.

**Writing – original draft:** Qing Zhou.

**Writing – review & editing:** Yu-Gen Chen.
